# Osthole alleviates neuropathic pain in mice by inhibiting the P2Y_1_-receptor-dependent JNK signaling pathway

**DOI:** 10.18632/aging.103114

**Published:** 2020-05-04

**Authors:** Ruili Li, Shajie Dang, Minna Yao, Chao Zhao, Wei Zhang, Jia Cui, Jingwen Wang, Aidong Wen

**Affiliations:** 1 Department of Pharmacy, Xijing Hospital, Fourth Military Medical University, Xi'an, Shaanxi 710032, China; 2 Department of Anesthesiology, Shaanxi Provincial Cancer Hospital, Xi’an, Shaanxi 71061, China

**Keywords:** neuropathic pain, osthole, P2YR _1_, JNK, EPSP

## Abstract

There are many reports about natural products relieving neuralgia. Osthole is the main component of *Angelica biserrata Yuan et Shan*, a natural product that treats rheumatism through the elimination of inflammation and the alleviation of pain that has a long history in the clinic. The analgesic mechanism of osthole is complicated and confusing. Astrocytes have attracted increasing attention from pain researchers. Inhibitors targeting astrocytes are thought to be promising treatments for neuropathic pain. Whether osthole can alleviate neuropathic pain through astrocytes has not been elucidated in detail. In this study, CCI surgery was used to establish the neuropathic pain model in mice. The CCI mice were treated with osthole (5, 10, or 20 mg/kg/day) for 14 days *in vivo*. Mechanical allodynia and heat hyperalgesia were measured to evaluate the therapeutic effect of osthole. In mechanism research, the activation of astrocytes; the protein expression of P2Y_1_R and p-JNK in astrocytes; the release of inflammatory factors; the variations in mEPSPs and eEPSPs; and the levels of GluA1, GluN2B, p-ERK, p-CREB and c-Fos in neurons were observed. The P2Y_1_R inhibitor MRS2179 and the p-JNK inhibitor SP600125 were used to demonstrate how osthole works in neuropathic pain. In addition, astrocytes and neurons were used to estimate the direct effect of osthole on astrocyte-neuron interactions and signal transmission *in vitro*. Our findings suggest that osthole treatment obviously relieved mechanical allodynia and heat hyperalgesia in CCI mice. P2Y_1_R is involved in CCI-induced pain hypersensitivity, and P2Y_1_R is required for osthole-induced p-JNK downregulation in the spinal cord. Osthole inhibited astrocyte activation and reduced inflammatory factor expression. After osthole treatment, mEPSP frequency and eEPSP amplitude were decreased in spinal lamina I-II neurons. Downstream signaling molecules such as pGluA1, pGluN2B, p-ERK, p-CREB and c-Fos were also reduced very quickly in osthole-treated neuralgic mice. Our conclusion is that osthole alleviates neuropathic pain in mice via the P2Y_1_-receptor-dependent JNK signaling pathway in spinal astrocytes, and osthole could be considered a potential pharmacotherapy to alleviate neuropathic pain.

## INTRODUCTION

Neuropathic pain inducing by a primary lesion or disease affecting the somatosensory system is a devastating disease which mainly manifested as hyperalgesia, allodynia and spontaneous pain, and comorbidities such as anxiety, depression and sleep disorder, which seriously affects patients’ quality of life and bring extreme financial burden to the country [1–3]. The theory and practice of pain treatment have long focused on neurons and designed therapeutic plan on these grounds. These therapeutic methods are not particularly effective because of various unconquerable side effects [4, 5]. It is necessary to explore deeply the surroundings in which neurons are exposed when pain occurs. In recent years, astrocyte, which have the power to regulate the functioning of neurons, have attracted more and more attention from pain researchers [6]. Astrocytes were activated by peripheral nerve injury, inflammation and tumors etc., which play a crucial role in development of neuropathic pain. Analgesic strategies targeting astrocyte provide a new way to cure neuropathic pain.

Astrocytes not only provide stable niches for signal transduction between neurons, but also act as immune response cells, producing a large number of inflammatory mediators, which make a further effect on astrocyte itself and bound to the corresponding receptors on neurons to enhance the excitability of neurons [7]. Following nerve injury, intracellular Ca^2+^ increased after ATP activated in extracellular fluid. Spinal astrocyte is activated by ATP through P2X and P2Y family receptors leading to release of inflammation cytokines (TNF-α, IL-1β and IL-6, et al.), chemokines (CXCL1, CCL-2, fractalkine, et al.) and neurotrophic factors [8]. P2Y_1_R is an important receptor in the sensory system, which expressed in astrocyte, microglia and neurons [9]. P2Y_1_R has emerged as the core astrocyte-neuron signaling pathway. It is reported that P2Y_1_R participated in pain process, promoting pain development. In formalin-induced inflammatory pain, P2Y_1_ receptor was increased in astrocyte [10]. In carrageenan injected rats, P2Y_1_R antagonist reduced IL-1β induced thermal hypersensitivity in a dose-dependent manner [11]. Similarly, P2Y_1_R antagonist prevents thermal hyperalgesia via inhibition of TRPV1 expression in dorsal root ganglion of neuralgia rat [12]. JNK, a family member of the MAPKs, was significantly activated in astrocyte of the spinal cord after nerve injury [13]. Therefore, JNK phosphorylation in astrocyte was considered as a reliability index which indicated astrocyte activation [14], and plays important roles in pain sensitization through regulating inflammatory cytokines and chemokines [15, 16]. P2Y_1_R activation takes part in different intracellular signaling pathways in astrocytes. A number of studies demonstrated that the P2Y1 receptor is in place upstream of the MAPKs and stimulate the prolonged activation of phosphorylation of JNK [17, 18]. Considering the importance of P2Y_1_ receptor-dependent JNK signaling pathway, we regard it as a potential target for the treatment of neuralgia.

Many natural medicines, especially those containing coumarins, have a pain relieving effect. There is a large number of scientific evidence manifesting the sturdy inhibitory effect of coumarins on pain [19–23]. In a prospective, randomized, placebo-controlled clinical trial, coumarin glycosides was used to relieve pain after stapled anopexy, and achieved good analgesic effect [24]. Our previous studies suggest that coumarins in the *Angelica biserrata Yuan et Shan*, a nature product to resist inflammation and alleviate pain in the loins and knee for a long history in clinic, relieved SNI induced neuralgia through TRPV1 in dorsal root ganglion [25]. Osthole, a coumarins, has been identified as a primary active constituent of the *Angelica biserrata Yuan et*. There are few reports on analgesia of osthole and the mechanism is mainly focused on the peripheral dorsal root ganglion [26]. It is necessary to conduct comprehensive study about mechanism protecting against neuropathic pain of osthole, especially the central mechanism in spinal and brain. P2Y_1_ receptor-dependent JNK signaling pathway in spinal astrocyte may be potential target of osthole in the treatment of neuralgia.

In the current study, we aimed to investigate the underlying mechanism of osthole treatment in neuralgia mice in spinal and determine whether P2Y_1_ receptor-dependent JNK signaling pathway in spinal astrocyte was involved in CCI induced neuropathic pain. We also explored whether pharmacological decreases in P2Y_1_R induced by osthole exerts an inhibitory effect on JNK. We expected that our research can provide more experimental basis for the application of osthole in clinic.

## RESULTS

### Osthole inhibits mechanical and thermal hyperalgesia in neuralgia mice

**Figure 1:**
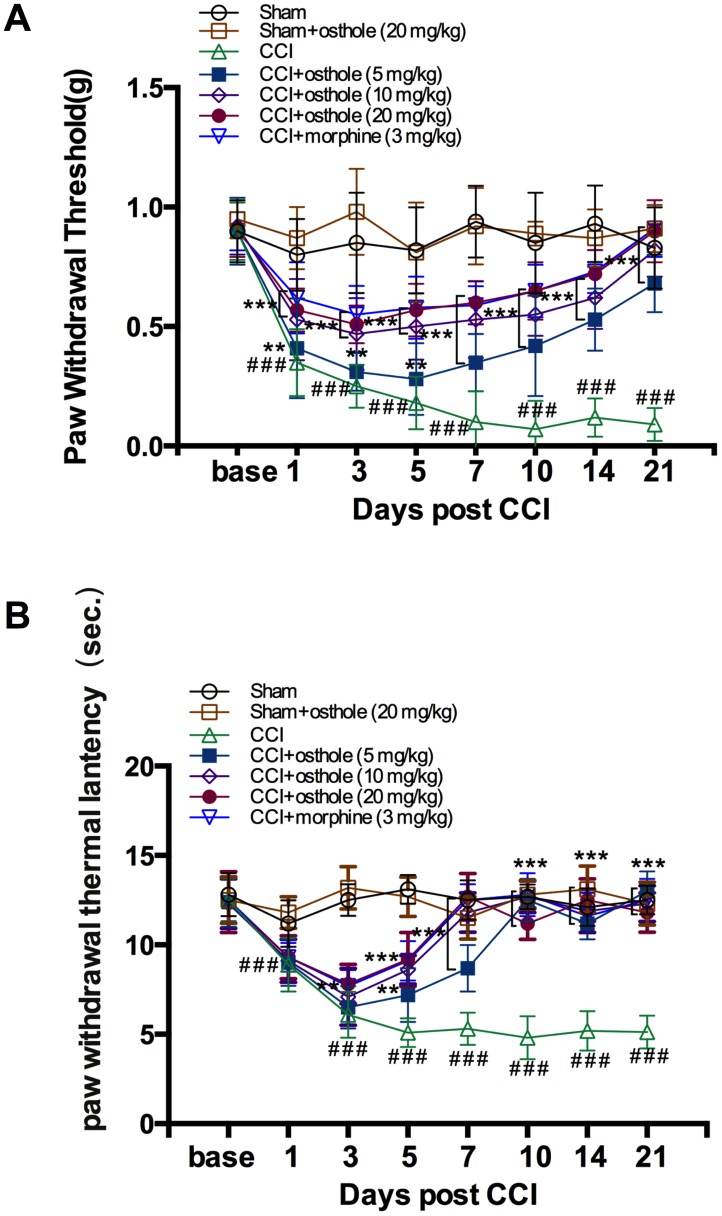
**Osthole attenuates CCI-induced mechanical and thermal hyperalgesia.** (**A**, **B**) Paw withdrawal threshold and Paw withdrawal latency were both significantly decreased on POD 3, maintained on POD 7. The pain threshold values of osthole-treated and morphine-treated groups were increased day by day, approaching the value of the sham group on POD 10. ###*p*<0.01, compared with sham group; **p<0.05, ***p<0.01, compared with CCI group, ANOVA followed by Bonferroni post-hoc test, n = 6 mice/group. All data are means ± SD.

A CCI-induced pain model was used to explore the effect of osthole or morphine on neurodynia and behavior was assessed on POD 0, 1, 3, 5, 7, 10, 14 and 21. Behavioral research revealed that CCI induced a seriously pain hypersensitivity with a characteristic of mechanical allodynia and heat hyperalgesia compared to sham group, maintaining for over 3 weeks (Figs. 1A, B). In sham mice, the ipsilateral hind paws showed no difference in PWT and PWL at all the time point. In CCI mice, PWT was significantly decreased on POD 3, maintained on POD 7 and decreased from 0.90 ± 0.12g on POD 0 to 0.09 ± 0.07g on POD 21(Figure 1A). PWL of CCI mice changed in a similar way and decreased from 12.50 ± 1.21s on POD 0 to 5.13 ± 0.92s on POD 21(Figure 1B). We detected the influence of osthole and morphine on CCI-induced pain. Osthole (5, 10 or 20 mg/kg) severely reduced mechanical allodynia and heat hyperalgesia continued for at least 3 hours with the best repression 1-hour post osthole treatment compared to CCI group. The pain threshold values of osthole treatment and morphine treatment groups were raised day by day, reaching the level of the sham group on POD 10. Although high dose osthole obviously cut down mechanical allodynia and heat hyperalgesia in CCI-mice, it did not change the baseline thresholds in sham group, indicating osthole has an important role for neuropathic pain conditions. Moreover, high dose osthole showed an equal pain threshold compared with that of the morphine treatment group after POD 10.

### CCI induced astrocytic P2Y_1_R up-regulation and JNK activation in the spinal cord

**Figure 2:**
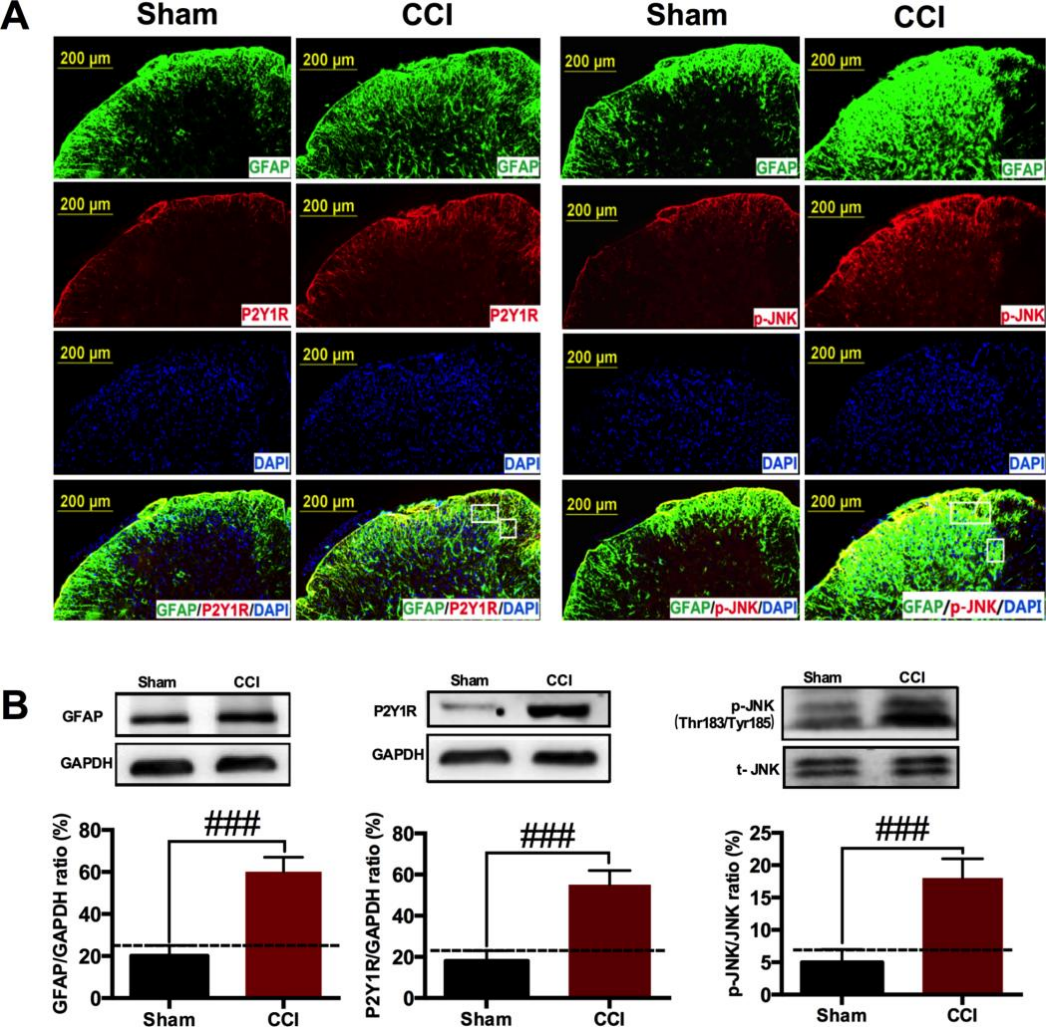
**CCI induced astrocytic P2Y_1_R up-regulation and JNK activation in the spinal cord.** (**A**) Immunostaining showing that CCI injury induced astrocytic marker GFAP expression in the ipsilateral spinal cord on POD 14. JNK phosphorylation and P2Y_1_R up-regulation occurred accompanied with astrocyte activation. Scale bar=200μm. (**B**) Western blot showing that GFAP, p-JNK and P2Y_1_R were increased in CCI group compared with sham group. All data are means ± S.D. ###*p*<0.01, compared with sham group; n=3 mice/group. The differences between groups were analyzed by ANOVA followed by Bonferroni post-hoc test.

Astrocyte activation is a common outcome of neuropathic pain. P2Y receptor family up-regulated and MAPKs activation in astrocytes are involved in neuropathic pain [27]. As shown in Figure 2A, CCI injury induced GFAP, astrocytic marker increased in spinal cord on POD 14. Meanwhile, P2Y_1_R up-regulation and JNK phosphorylation occurred accompanied with astrocyte activation. Double staining showed that P2Y_1_R and p-JNK in astrocytes promote the development of neuropathic pain. The western blotting results further reflect that GFAP, P2Y_1_R and p-JNK protein expression were increased in CCI group compared to sham group (Figure 2B), indicating that a P2Y_1_R-dependent JNK pathway was drawn into CCI-induced neuralgia.

### Osthole cut down P2Y_1_R up-regulation and p-JNK activation on a concentration-dependent manner

**Figure 3:**
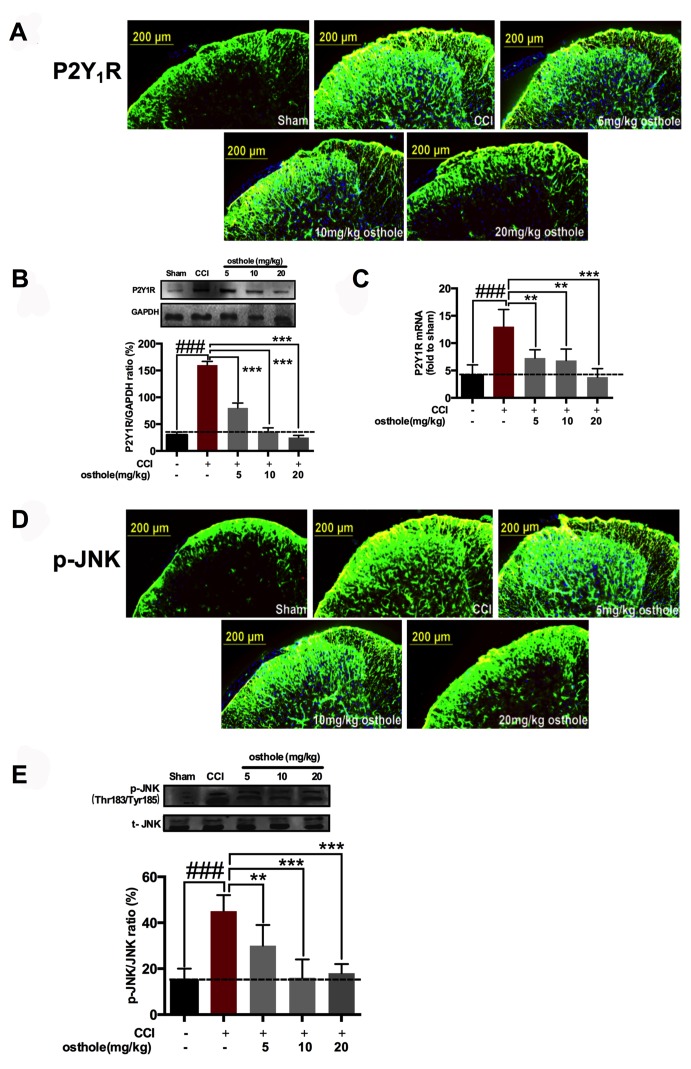
**Osthole cut down P2Y_1_R up-regulation and p-JNK activation on a concentration-dependent manner.** (**A**–**C**) Osthole decreased P2Y_1_R of CCI mice on a concentration dependent manner. ###p<0.01, compared with sham group; **p<0.05, ***p<0.01, compared with CCI group, ANOVA followed by Bonferroni post-hoc test, n=6 mice/group. (**D**, **E**) Osthole reduced p-JNK expression after CCI in the spinal cord on POD 14. ###p<0.01, compared with sham group; **p<0.05, ***p<0.01, compared with CCI group, ANOVA followed by Bonferroni post-hoc test, n=6 mice/group. (**F**) Osthole reduced inflammatory factors after CCI in the spinal cord on POD 14. ###*p*<0.01, compared with sham group; ***p<0.01, compared with CCI group, ANOVA followed by Bonferroni post-hoc test, n=6 mice/group. All data are means ± S.D.

We harvested the spinal cords of mice treated with osthole (5, 10 or 20 mg/kg) on POD 14. Immunostaining showed that P2Y_1_R immunoreactivity was decreased by osthole. In particular, P2Y_1_R immunoreactivity treated with 20 mg/kg osthole was similar to those observed for sham group (Figure 3A). Western blot analysis showed that osthole decreased P2Y_1_R of CCI mice on a concentration dependent manner (Figure 3B). Osthole treatment also attenuated P2Y_1_R mRNA compared to CCI group (Figure 3C). Meanwhile, Immunostaining showed that osthole could decrease the immune response of p-JNK. In particular, p-JNK immunoreactivity treated with 20 mg/kg osthole was nearby to that seen in sham group (Figure 3D). Western blot analysis showed that osthole decreased p-JNK of CCI mice on a concentration dependent manner (Figure 3E). Furthermore, our results showed that osthole treatment attenuated TNF-α, IL-1β, and IL-6 expression on a dose dependent manner (Figure 3F) in the spinal cord. The results indicated that P2Y_1_R and p-JNK participated in CCI-induced pain, osthole attenuated P2Y_1_R up-regulation and p-JNK activation on a concentration-dependent manner.

### Osthole inhibited neuronal excitability and excitatory synaptic transmission

**Figure 4:**
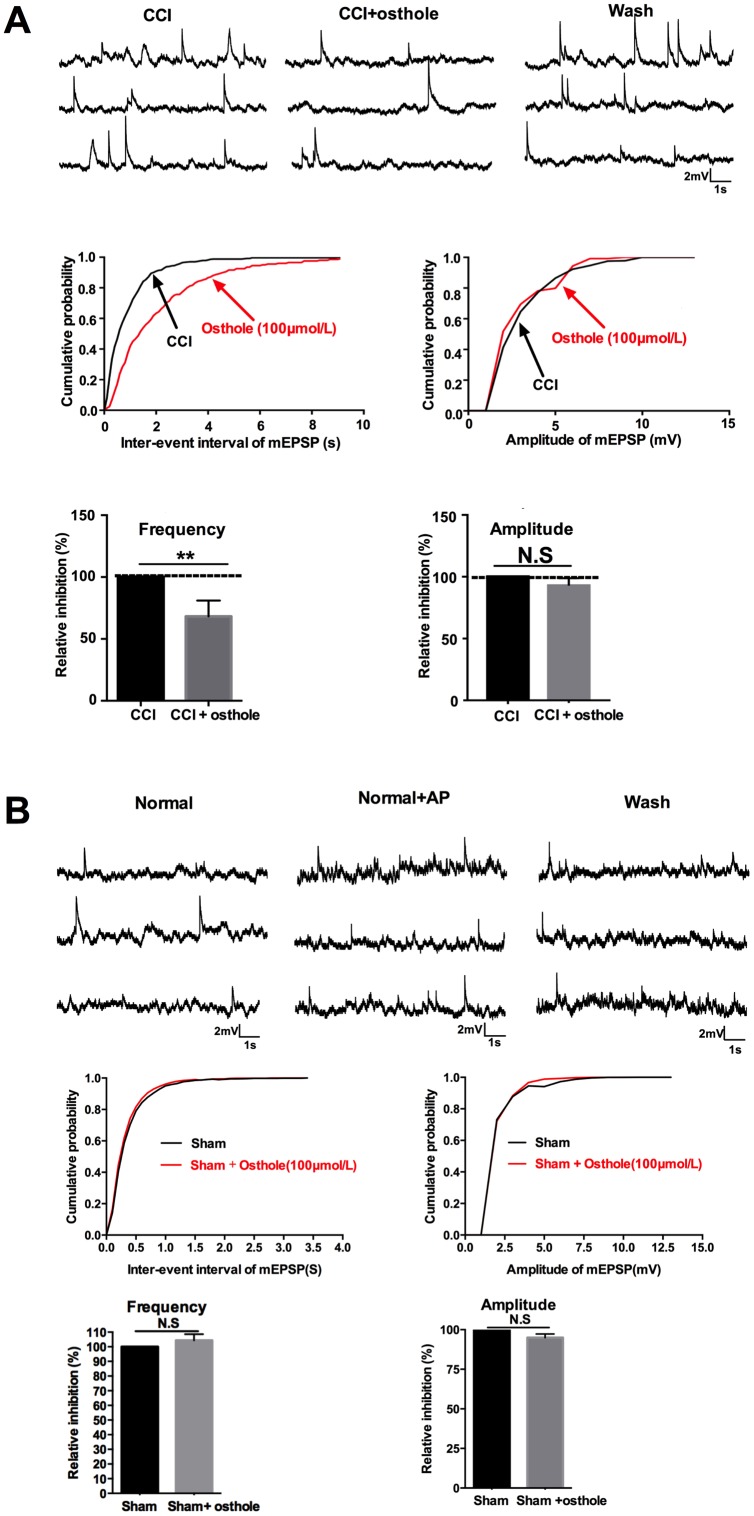
**Osthole induced synaptic inhibitory effects.** (**A**) mEPSPs frequency was depressed by 100 μmol/L osthole, but mEPSPs amplitude did not show significant changes. (**B**) Osthole did not alter mEPSPs frequency and amplitude of normal mice. pCLAMP 10 software (Axon Instruments) were used to acquire and analyze the potential data. **p<0.05, compared with CCI group, ANOVA followed by Bonferroni post-hoc test, n = 6 mice/group. All data are means ± SD.

**Figure 5:**
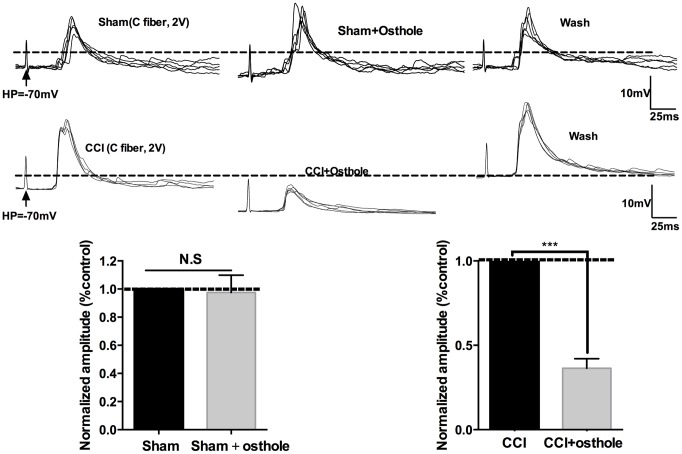
**Osthole inhibits excitatory synaptic transmission.** Traces of eEPSPs in lamina 1-II neurons in the presence of osthole. The histogram indicates the monosynaptic eEPSP peak amplitude compared with control. ***p<0.01, compared with CCI group, ANOVA followed by Bonferroni post-hoc test, n = 6 mice/group. All data are means ± SD.

Signals deliver of nervous system is inseparable with synaptic transmission. Neuronal excitability represents the severity of neuralgia. In order to explore the effect of osthole on neuronal excitability, mEPSPs of lamina I-II neurons in CCI mice were detected with or without osthole perfusion. As shown in Figure 4A, mEPSPs frequency was depressed by 100 μmol/L osthole, but mEPSPs amplitude did not show significant changes. Furthermore, we checked mEPSPs of lamina I-II neurons of normal mice with or without osthole treatment. Figure 4B showed that osthole did not alter mEPSPs frequency and amplitude of normal mice. These findings suggest that osthole effectively inhibit excitatory synaptic transmission. We also tested the actions of osthole on excitatory synaptic inputs to neurons in lamina I-II from C fibers. As shown in Figure 5, a reproducible monosynaptic eEPSPs in the lamina I-II neurons was evoked. Osthole bath-applied for 10 minutes at a dose of 100 μmol/L. The amplitude of C fiber-evoked monosynaptic eEPSPs were significantly decreased by osthole compared with CCI group.

### Osthole inhibited neuronal cell signaling molecules in a dose dependent manner

**Figure 6:**
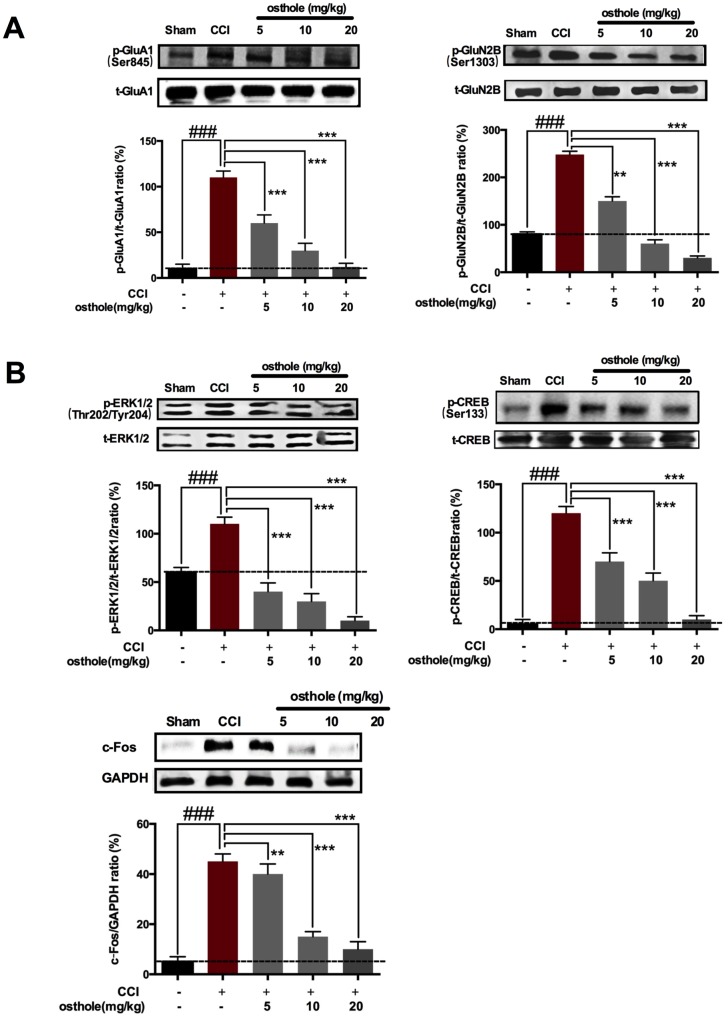
**The effect of osthole on Postsynaptic receptor pain-related signal molecules in neurons of CCI mice.** (**A**) pGluA1 (ser845) and pGluN2B (S1303) were decreased after osthole treatment compared to CCI mice. (**B**) Osthole dose-dependently inhibited expression of p-ERK, p-CREB and c-Fos protein compared to CCI group. ###*p*<0.01, compared with sham group; **p<0.05, ***p<0.01, compared with CCI group, ANOVA followed by Bonferroni post-hoc test, n = 6 mice/group. All data are means ± SD.

When inflammation cytokines and chemokines bind to their receptor, what happens in neuron as follows? Glutamate in presynaptic element liberation increased, causing glutamic acid receptor in postsynaptic element activated, such as AMPA and NMDA. Excitatory receptors in neurons leads to increased calcium influx [28], initiating a series of downstream signal pathways, including p-ERK, p-CREB and c-Fos, which are closely related with pain sensitivity [3]. Postsynaptic receptor was also detected to further prove the influence of osthole on neuronal excitability. The results showed that pGluA1 (ser845) and pGluN2B (S1303) were decreased after osthole treatment compared to CCI mice (Figure 6A). At the same time, pain-related signal molecules in neurons of CCI mice were checked. We found that CCI induced p-ERK, p-CREB and c-Fos increased compared to sham group, while osthole dose-dependently inhibited expression of p-ERK, p-CREB and c-Fos protein compared to CCI group (Figure 6B). In summary, osthole induced synaptic inhibitory effects and reduced neuronal excitability.

### P2Y_1_ receptor-dependent JNK signaling pathway in spinal astrocyte was involved in pain relief after osthole treatment

**Figure 7:**
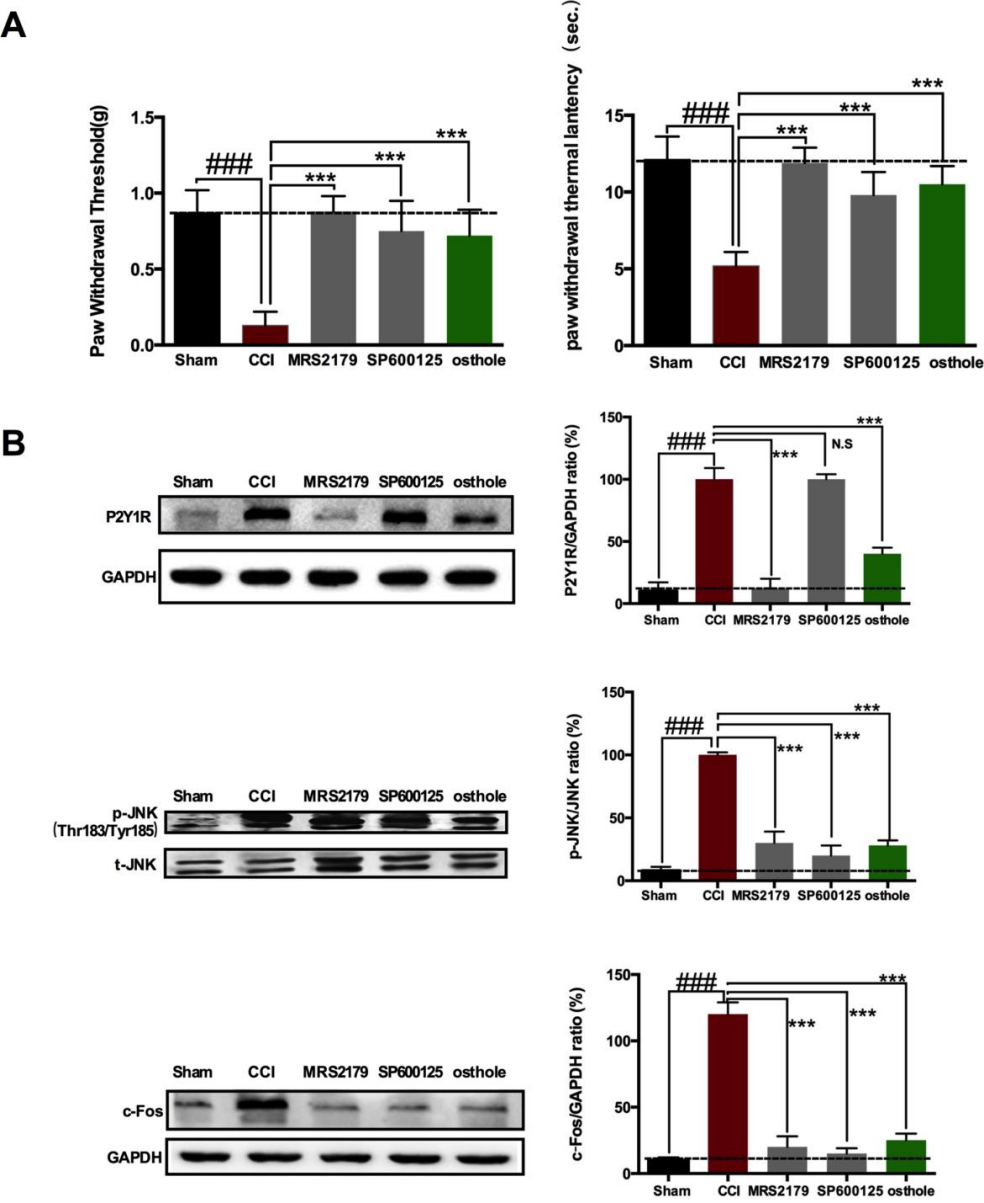
**P2Y_1_ receptor-dependent JNK signaling pathway in spinal astrocyte was involved in pain relief after osthole treatment.** (**A**) Single injection MRS2179, SP600125 and osthole all seriously changed the CCI-induced mechanical allodynia and heat hyperalgesia. (**B**) The effect of MRS2179, SP600125 and osthole on P2Y_1_R, p-JNK and c-Fos expression compared to CCI group. ###*p*<0.01, compared with sham group; ***p<0.01, compared with CCI group, ANOVA followed by Bonferroni post-hoc test, n=6 mice/group. All data are means ± S.D.

Research shows that when neuropathic pain occurred, c-Fos expression increased. c-Fos can be used as a marker of neuropathic pain. A dose of 4 μg P2Y_1_R inhibitor MRS2179, 5 μg p-JNK inhibitor SP600125 or 10mg/kg osthole were used to explore molecular mechanism of osthole in pain relief and clarify the relationship between P2Y_1_R, osthole and c-Fos. Behavior was assessed four hours after MRS2179, SP600125 or osthole was injected on POD 14. As shown in Figure 7A, single injection MRS2179, SP600125 and osthole all seriously changed the CCI-induced mechanical allodynia and heat hyperalgesia. The pain threshold values of MRS2179, SP600125 and osthole treated group were increased compared to CCI group. We harvested the spinal cords of mice treated with MRS2179, SP600125 or osthole on POD 14. Western blot analysis demonstrated that MRS2179 and osthole significantly depressed P2Y_1_R, p-JNK and c-Fos expression compared to CCI group. Interestingly, SP600125 significantly depressed p-JNK and c-Fos expression, while has no effect on P2Y_1_R (Figure 7B). These results show that JNK signaling pathway adjusted by P2Y_1_R, osthole exerts analgesic effect through P2Y_1_R.

### Osthole exerts analgesic effect by directly acting on P2Y_1_ receptor of astrocyte

**Figure 8:**
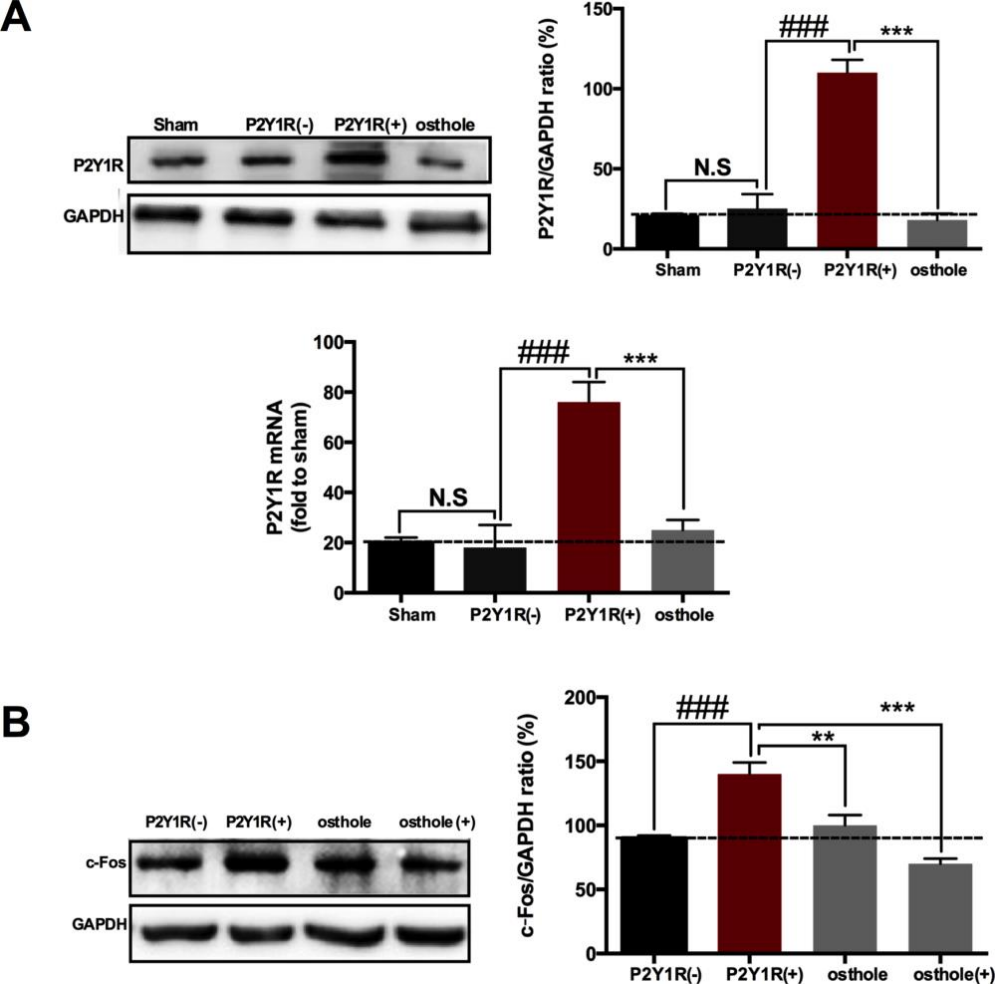
**Osthole exerts analgesic effect by directly acting on P2Y_1_ receptor of astrocyte.** (**A**) P2Y_1_R expression in transfected cells markedly increase compared to normal astrocyte. (**B**) Astrocyte-conditioned medium induced the change trend of c-Fos consistent with that of P2Y_1_R. ###*p*<0.01, compared with sham group; ***p<0.01, compared with P2Y_1_R overexpression group, ANOVA followed by Bonferroni post-hoc test, n=6 mice/group. All data are means ± S.D.

To prove the effect of osthole on P2Y_1_R, primary astrocytes transfect with a pcDNA3.1 vector contained P2Y_1_R sequence. Our results showed that P2Y_1_R sequence was successfully transfected into astrocyte, P2Y_1_R expression in transfected cells markedly increase compared to normal astrocyte. Meanwhile, osthole obviously decreased P2Y_1_R protein and gene levels (Figure 8A). We also detected c-Fos expression in primary neurons dealt with astrocyte-conditioned medium. Our research showed that astrocyte-conditioned medium induced the change trend of c-Fos consistent with that of P2Y_1_R. In astrocyte-conditioned medium pretreated with P2Y_1_R (+), expression of c-Fos significant increase and attenuated in osthole group. Meanwhile, c-Fos expression decreased further in osthole (+) group (Figure 8B). Data above indicated that the effect of osthole on P2Y_1_R in astrocyte leads to decrease of c-Fos expression in neurons and produces analgesic effect. Perhaps, osthole has some direct effect on neurons.

## DISCUSSION

The present study reported that osthole present chronic analgesic effect in a mice model of CCI induced neuropathic pain. For the first time, we studied in detail how does astrocyte participate in the analgesic effect of cells on the mediation of neurons. Our findings showed that osthole treatment obviously relieved the mechanical allodynia and heat hyperalgesia in CCI mice. P2Y_1_R partake in CCI-induced pain, and P2Y_1_R is essential for osthole-induced JNK phosphorylation down-regulation in spinal cord. Osthole inhibited astrocyte activation and reduced inflammatory factors expression. Meanwhile, EPSPs and downstream signaling molecule related pain was also reduced. Osthole could be considered as a potential pharmacotherapy to alleviate neuropathic pain. To study the analgesic mechanism of osthole is helpful for its clinical promotion.

Astrocyte activation is closely associated with the development of persistent pain following nerve injury. Analgesic effects can be produced by inhibiting astrocyte activation or effectively antagonizing the pain-relieving substances released by astrocyte. After peripheral nerve injury, astrocytes in the dorsal horn of spinal cord are usually activated through noxious neurotransmitters such as ATP, glutamate, SP and calcitonin-gene-related peptide released from terminals of primary neurons or pro-inflammatory factors (such as IL-1β, IL-6, TNF-α and IL-18) [29]. As an important exogenous substance, ATP activated astrocyte through P2X and P2Y family receptors (such as P2X_4_, P2X_7_ and P2Y_6_ in Microglia, P2Y_1_ and P2Y_2_ in neurons and P2Y_1_ and P2Y_11_ in astrocyte) [30]. Astrocyte activation is characterized by GFAP up-regulation and signal molecules phosphorylation [31]. Based on the characteristics, Scientists have done a lot of research on astrocytes and the molecules they express after activation [32, 33]. In the current study, we proved that CCI injury induced astrocytic marker GFAP increased in spinal astrocyte on POD 14. Firstly, we checked the role of P2Y_1_R in the relief of neuralgia by osthole. The results showed that P2Y_1_R up-regulation occurred accompanied with astrocyte activation. Osthole decreased P2Y_1_R of CCI mice on a concentration dependent manner. P2Y_1_R inhibitor obviously reduced the CCI-induced mechanical allodynia and heat hyperalgesia. The pain threshold values of P2Y_1_R inhibitor MRS2179 treated group were increased compared to CCI group. These results are supported by the viewpoint of protein kinase cascades were able to modulate P2Y and P2X receptor [34], and coumarin was a protein Kinase Inhibitor [35]. The relationship between osthole, protein kinase cascades and P2Y_1_R need to be further confirmed by other experiments. Secondly, we examined the effect of osthole on p-JNK in the treatment of neuralgia. We proved that JNK phosphorylation occurred accompanied with astrocyte activation. Osthole decreased p-JNK of CCI mice on a concentration dependent manner. The pain threshold values of p-JNK inhibitor SP600125 treated group were increased compared to CCI group. Thirdly, we explored the relationship between P2Y_1_R and p-JNK in the treatment of neuralgia with osthole. Interestingly, osthole and MRS2179 could reduce the expression of p-JNK protein, while SP600125 has no effect on P2Y_1_R, indicating p-JNK is a downstream molecule of P2Y_1_R, which expression is regulated by P2Y_1_R. Meanwhile, osthole attenuated overexpression of P2Y_1_R protein in culture astrocyte, indicating osthole exerts analgesic effect by directly acting on P2Y_1_R of astrocyte. Overall, we firstly revealed the relationship between P2Y_1_R and p-JNK in neuralgia, and how osthole act on P2Y_1_ receptor-dependent JNK signaling pathway in neuralgia mice.

Inflammatory factors and chemokines, released in astrocyte, bind to specific receptor in neurons and increase neuronal excitability [36]. Simultaneously, synaptic transmission of AMPA and NMDA receptor raised, and AMPA and NMDA expression increased on neuron cell membrane. Presynaptic release of substance P and other peptides can induce slow depolarization, resulting in NMDA receptor containing NR2 (A-D) subunit opened and AMPA receptor containing GluR1 subunit increased. Therefore, the inward current is enhanced and excitatory synaptic transmission of LTP is enhanced to promote the spread of pain [37]. In the current study, we found that osthole treatment attenuated TNF-α, IL-1β, and IL-6 expression on a dose dependent manner in CCI mice. Osthole treatment decreased the mEPSPs frequency and eEPSPs amplitude of CCI mice, reduced p-GluA1 and p-GluN2B expression in the spinal cord of CCI mice. When recording eEPSPs, strychnine and bicuculline were used to block the inhibitory transmitters. Previous findings suggest that mEPSPs frequency mediated presynaptic effect and mEPSPs frequency amplitude mediated postsynaptic [37]. Accordingly, osthole reduced neuronal excitability of CCI mice by presynaptic mechanism. However, postsynaptic receptors such as p-GluA1 and p-GluN2B were also decreased by osthole in neuralgia mice on POD 14. We speculate that osthole reduced neuronal excitability of CCI mice by not only presynaptic but also postsynaptic mechanism. That is because time is too short to transcribe and translate postsynaptic receptors under osthole perfusion condition. If possible, we will record mEPSPs of lamina I-II neurons in spinal cord of CCI mice on POD 14. In our research, p-GluA1 and p-GluN2B increase were observed in CCI mice, which was different with viewpoint of Ho et al. [38] that SNL only induced NR1 and NR2B subunits up-regulated. Excitatory synaptic transmission promotes Ca^2+^ enters postsynaptic neurons, leading to activation of a series of signaling pathways and central sensitization. ERK pathway plays a key role in neuronal plasticity and central sensitization. ERK phosphorylation activates transcription factor CREB. CREs, as promoter, encode pain-related genes such as c-Fos, COX-2, NK-1, dynorphin and TrkB [39]. Our findings are consistent with previous research. Osthole dose-dependently inhibit p-ERK, p-CREB and c-Fos in the spinal cord of CCI mice. C-Fos is often used as a marker of pain [46]. Single injection MRS2179, SP600125 and osthole could all reduce the expression of c-Fos protein. In culture neurons treated with astrocyte-conditioned medium, osthole further reduce c-Fos expression, indicating osthole has a direct effect on neurons.

The findings support the viewpoint that osthole alleviates neuropathic pain in mice via P2Y_1_ receptor-dependent JNK signaling pathway in spinal astrocyte, and it could be considered as a potential pharmacotherapy to alleviate neuropathic pain. Astrocyte-neuron interaction extremely complicated, involved in multiple signal molecules. Our study clearly elucidates the mechanism of osthole treatment of neuralgia at the spinal cord level, but there are still many shortcomings. Coumarins possess potent estrogen-like effect, which remedy neuralgia and depression directly regulate NMDA receptor [40, 41]. The direct effect of osthole on NMDA receptor has not been reported and need further research. Furthermore, adding osthole to spinal cord slices altered both mEPSP and eEPSP recorded from lamina I-II neurons within 10 min. This would implicate direct effect on neurons. Cell experiments also hint at this idea. This is because P2Y_1_R exit not only in astrocyte, but also in neurons and microglia. In other experiment, we will study how osthole works on neurons and microglia to relieve pain in details. Anyway, it can be assured that P2Y_1_ receptor-dependent JNK signaling pathway in astrocyte was involved in the process of treating neuralgia with osthole. Our research revealed analgesia mechanism of osthole, providing a reliable basis for its clinical application.

In conclusion, the results of the present study indicated that osthole treatment obviously relieved mechanical allodynia and heat hyperalgesia in CCI mice. Osthole alleviated neuropathic pain in mice via the P2Y_1_-receptor-dependent JNK signaling pathway in spinal astrocytes. Furthermore, osthole reduced neuronal excitability through the astrocytic P2Y_1_-receptor-dependent JNK signaling pathway in spinal astrocytes in neuralgic mice.

## MATERIALS AND METHODS

### Drug and reagents

Osthole (>98%, CAS: 484-12-8) was obtained from Yuanye Biotechnology Co., Ltd. (Shanghai, China). Mouse Anti-GFAP (GA5), Rabbit Anti-SAPK/JNK, Rabbit Anti-phospho-ERK (1/2) (Thr202/Tyr204), Rabbit Anti-ERK (1/2), Rabbit Anti-phospho-CREB, Rabbit Anti-CREB and Rabbit Anti-c-Fos (9F6) were obtained from Cell Signaling Technology (Danvers, United States). Rabbit Anti-phospho-JNK (Thr183/Tyr185), Mouse Anti-phospho-Glutamate Receptor 1(AMPA subtype) (S845), Rabbit Anti-Glutamate Receptor 1(AMPA subtype), Rabbit Anti- phospho-NMDAR2B (s1303), Rabbit Anti-NMDAR2B and Rabbit Anti-GAPDH were obtained from Abcam technology (Cambridge, United Kingdom). Rabbit Anti-P2Y_1_R for immunofluorescence was obtained from Yansheng technology (Shanghai, China). Goat Anti-Rabbit IgG and Goat Anti-Mouse IgG for western blots and Immunofluorescence were all purchased from Cell Signaling Technology (Danvers, United States). TNF-α, IL-1β and IL-6 ELISA kits were provided by Abcam technology (Cambridge, The United Kingdom). DAPI dihydrochioride was purchased from Diyi technology (Shanghai, China). MRS2179 and SP600125 were purchased from Abcam technology (Cambridge, The United Kingdom). DMEM medium is got from HyClone (Logan, USA). Lipofectamine 2000 was purchased from Thermo Fisher Scientific (Waltham, USA). Trizol reagent and SYBR Premix Ex TaqTM II kit were purchased from TaKaRa (Shiga, Japan). All other reagents are analytical reagents and are available on the market.

### Animals and experimental design

C57BL/6 mice, 18~22 g, were obtained from the Experimental Animal Center of Fourth Military Medical University. Under a 12/12 h light/dark cycle, mice were placed in controlled indoor humidity (45~75%) and temperature (22±2°C) and provided water and food at will. Mice were acclimatized for 4 days to the laboratory conditions and for 3 days to manipulations and devices prior to behavioral studies [42]. This experiment was carried out with consent of Chinese Food and Drug Administration (cFDA). Best effort was contributed to minimize the number of animals used and their suffering.

To investigate the analgesic effect of osthole in neuropathic pain, seven groups of animals were used: sham group, treatment of sham with osthole (20 mg/kg), CCI model treated with osthole (0, 5, 10, or 20 mg/kg) and morphine (3 mg/kg) groups. The sham group and CCI group received a same volume of vehicle. Conduct a behavioral experiment on post-operative day (POD) 0, 1, 3, 5, 7, 10, 14 and 21 every day after surgery. Osthole was administered intraperitoneally after surgery from POD 0 to POD 14. On POD 14, mice were killed for experiment. MRS2179 and SP600125 were injected to mice intrathecal pathway and transported to the cerebral spinal fluid in a 30G needle with a total volume of 20 μl between the L5 and L6 intervertebral. To investigate the mechanism of osthole in cultured cells, the treatment of osthole (100 μmol) was started 24 h. MRS2179 and SP600125 were started 1 h before cells collected.

### CCI induction in mice

The well-established L5-L6 CCI model of neuropathy was used in this study. Briefly, mice were anesthetized with 1% pentobarbital (10 mL/kg) intraperitoneally (i.p.), and the left common sciatic nerve of each mouse was exposed at the mid-thigh level. Close to the end of the sciatic trigeminal nerve, the nerve was separated from adhered tissue, and 4 ligatures (5-0 chronic gut) are loosely tied around it, about 1 mm between ligatures. After operation, the skin layers and muscle were sutured and iodine was used to disinfect the operation area [43].

### Behavioral assessments of mechanical allodynia and heat hyperalgesia in mice

All manipulations were performed under quiet conditions by the same experimenter in a test room to avoid stress. Use the calibrated von Frey filaments (Stoelting, Kiel, WI, USA) to assess claw contraction for mechanical stimulation. Animals were placed on an elevated mesh grid and habituated to the testing environment for at least 0.5 h before examination. A series of von Frey filament with logarithmical incremental stiffness (0.016~1g, Woodland Hills, Los Angeles) were used to stimulated the plantar surface of each hind in an order of increasing stiffness for 5 s. A positive response was indicated by rapid pulling back, biting, or shaking of the hind limb within 5 s of the application of von Frey filament. The interval between different filaments was at least 5 minutes. A single filament stimulated the same hind limb for 10 times. The minimum value that resulted in at least six responses to ten stimulations was recorded as the paw withdrawal threshold [44].

Paw withdrawal in response to noxious thermal stimuli was assessed using a Full-Automatic Plantar Analgesia Tester (BME-410C, Institute of biomedical engineering, CAMS) that produces radiant heat by directing a beam of light to the plantar surface of hind paw. The mice were placed in plastic boxes on a glass plate for at least 0.5 h. Six trials on the ipsilateral paw were performed with an interval of at least 5 min. To prevent tissue damage, radiant heat was administered for a maximum of 30 s.

### Spinal cord slices preparation and patch-clamp recording

Mice were anesthetized with 1% pentobarbital (10 mL/kg) intraperitoneally (i.p.) and perfused through the aorta with sucrose- substituted artificial cerebral spinal fluid (mmol/L: sucrose, 75.00; NaCl, 80; KCl, 2.5; CaCl_2_, 2.5; MgCl_2_, 1.2; NaH_2_PO_4_, 1.25; NaHCO_3_, 25; ascorbate, 1.3; and pyruvate, 3) aerated with 95% O_2_ and 5% CO_2_ for 30 minutes and stored at 4°C). The lumbar segment was dissected and fixed in sucrose ACSF quickly. The parasagittal spinal slice (450-500 μm thick) with an attached dorsal root (0.8-1.0 cm long) was cut with a vibrating microtome and was incubated with Glucose ACSF (mmol/L: NaCl, 125; KCl, 2.5; CaCl_2_, 2; MgCl_2_, 1; NaH_2_PO_4_, 1.25; NaHCO_3_, 26; d-glucose, 25; ascorbate, 1.3; and pyruvate, 3;) aerated with 95% O_2_ and 5% CO_2_) at room temperature (22°C-25°C, 3.5 mL/min) [45]. The procedures for patch-clamp recordings was as follows [46]. A pipette electrode (5-10MΩ), with Axon 200B enlarger was used to record mEPSPs of C fibers in lamina I-II neurons of spinal cord. In current-clamped mode, the potential was maintained at -50 mV to show mixed excitation and inhibitory synaptic events. For eEPSPs, the average amplitude was obtained before 5 minutes, during 10 minutes and after 20 minutes osthole application. In an intro hyper-excitability model (mmol/L: bicuculline, 10; strychnine, 2; Tetrodotoxin, 5), mEPSPs were recorded before 5 minutes, during 10 minutes and after 20 minutes osthole application.

### Primary cell culture

Astrocytes cultures were prepared from cerebral cortex of neonatal C57BL/6 mice. Neonatal mice were decapitated and meninges were carefully removed from isolated cerebral hemispheres in cold PBS. The cerebral hemisphere is separated into a separate cell suspension and collected by dissociation for 5 minutes at 400 g. The dissociated cells were suspended in the DMEM supplemented with 10% (v/v) FBS, penicillin (100 U/ml) and streptomycin (100 μg/ml). After trituration, the cell suspension was plated into 6-well plates at a density of 3×10^5^ cells/cm^2^, and cultured for 10-12 days [47]. The medium was changed twice a week. The fairly pure astrocytes (>90%) were prepared by shaking the flasks 180 rpm overnight and then culturing them with 10 ml of 0.05% trypsin in a cell incubator for 15 min to separate microglial cells and oligodendrocytes. Astrocytes were divided into 4 groups: Sham (Normal cultured cells), P2Y_1_R (-) (cells transfected with an empty pcDNA3.1 vector), P2Y_1_R (+) (cells transfected with a pcDNA3.1 vector contained P2Y_1_R sequence) and osthole (cells transfected with a pcDNA3.1 vector contained P2Y_1_R sequence and treated with 100μmol/L osthole for 24 h). Lipofectamine 2000 was used to transfect astrocytes. For neuron culture, the cell suspension was plated into a plate pre-coated with cell adherent reagent. After incubation in DMEM containing 10% FBS for 6 h, the medium was changed to B27 supplement and 0.5 mmol/L glutamine. The experiment started 5 to 6 days after electroplating. Harvested neurons showed dendritic spine morphology and exhibited about 90% purity. Astrocyte-conditioned medium pre-treated with P2Y_1_R (-), P2Y_1_R (+), osthole and osthole (+) (cells treated with 100μmol/L osthole directly) were used to substitute for half of the neuronal medium.

### Immunofluorescence

Mice were deeply anesthetized with 1% pentobarbital (10 mL/kg) intraperitoneally (i.p.), and perfused through the aorta with 20 mL of saline, then there is 50mL 4% paraformaldehyde. The spinal cord segment (L4-L5) was removed and frozen in 30% sucrose solution after being fixed overnight in 4 % formaldehyde. Spinal cord was cut into 20 μm-thick serial sections using a cryostat (Leica CM1950) and mounted on 3-aminopropyl-triethoxysilane-coated glass slides. Spinal cord sections were stored at −20 °C. Immunofluorescence was performed as follows. The sections were air-dried and circled with an Immunity Staining Guard Pen. Firstly, the slices were incubated with 3% bovine serum albumin containing 0.4% Triton X-100 acted at room temperature for 1 h. For dual immunofluorescence, the mixture of polyclonal and monoclonal primary antibodies were incubated overnight at 4 °C as follows: Phospho-JNK antibody (1:100, rabbit), P2Y_1_R antibody (1:500, rabbit), and GFAP antibody (1:500, mouse). The sections were washed in PBS and then incubated in the dark for 2 hours at room temperature. The secondary antibodies (1:1000) coupled with FITC were used to incubate sections at room temperature for 2 hours. Sections were washed 3 times for 5 min with PBS and incubated using a PBS-based mounting medium containing 10 μg/mL DAPI for 15 min. Sections were washed 3 times for 5 min with PBS and cover slipped using mounting liquid. Stained sections were visualized, and photographs were obtained under a fluorescent microscope (Leica Microsystems GmbH, Mannheim, Germany) [48].

### Western blot

Mice were anesthetized with 1% pentobarbital (10 mL/kg) intraperitoneally (i.p.) and the spinal cord segment of L4-L5 was removed. For cultured astrocytes and neurons, after incubation with osthole and other regents, cells were collected. The tissue or cell was homogenized in a radio immunoprecipitation assay lytic buffer containing 0.1 mol/L phenylmethyl sufonyl fluoride on ice for 30 min. Protein concentrations of each samples were determinate by bicinchoninic acid Protein Assay. Equal content of proteins (30 μg) was mixed with a one-fifth volume of 5× loading buffer and heated to 100°C for 5 min. Protein samples and rainbow-colored protein molecular markers were separated by 10% SDS- polyacrylamide gel electrophoresis gel and transferred to polyvinylidene fluoride membranes. In a Tris-buffered saline containing 0.5% Tween-20 solution, these blots were first sealed for 30 min and then incubated overnight at 4°C with antibody against GFAP (1:1000, mouse), P2Y_1_R (1:1000, rabbit), p-JNK (1:1000, rabbit), JNK (1:1000, rabbit), pGluA1 (1:1000, rabbit), GluA1 (1:1000, rabbit), p-GluN2B (1:1000, rabbit), GluN2B (1:1000, rabbit), p-ERK (1:1000, rabbit), ERK (1:1000, rabbit), p-CREB (1:1000, rabbit), CREB (1:1000, rabbit), and c-Fos (1:1000, rabbit). In order to control the load, the blots were probed with GAPDH antibody (1:1000, mouse). The Membrane is rinsed with TBST for 30 minutes, then cultured with horseradish peroxidase-conjugated secondary antibody for 2 h, and developed in electrochemiluminescence solution [49]. The intensity of protein bands was quantified using densitometry in Image ProPlus 6.0 software.

### Enzyme-linked immunosorbent assay

Mice were decapitated under anesthesia with an over-dose of 1% pentobarbital sodium. The plasma collected before animals sacrificed, homogenized L4-L5 spinal cord tissue, and the supernatant of astrocytes were all used to determine TNF-α, IL-1β, and IL-6 levels by corresponding ELISA kits. Absorbance was read at 450 nm. For each reaction in the 96-well plate, a 100 ml of sample was used and ELISA was performed in a three-link manner according to the manufacturer's instructions. The experiment includes a standard curve.

### Real-time quantitative PCR

Mice were deeply anaesthetized with pentobarbital sodium. Trizol reagent was used for the isolation of total RNA from L4-L5 spinal segments. RNA quantity and purity were determined using an ultra micro-volume spectrophotometer (NanoVue, General Electric Company, USA). cDNA is amplified by the following primers: P2Y_1_R forward, 5’-GAC TTC TTG TAC GTG CTG ACT CT-3’, P2Y_1_R reverse, 5’-GAC CTC TTG TCA CCT GAT ACG TG-3’; product size: 647 bp. All quantitative PCR reactions were performed in a Real-time Detection System (Applied Biosystem, USA) by QuantiTect Rev. Transcription Kit. PCR was amplified at 95°C for 30s, then 45 cycles at 95°C for 5s, 56°C for 30s, and 72°C for 30 s. Step-one Analysis Software is used to analysis data. The melting curves is obtained after the cycle is completed to ensure that there are no non-specific products.

### Statistical analysis

The data obtained were recorded as means ± S.D. Adult C57BL/6 mice (weighing 18~22 g) were randomly separated for behavioral studies (n=6) or biochemical studies (n=6), and n refers to the number of animals per group. One-way AVOVA were used to test the changes of values for the verification of significance. The differences between groups were analyzed by one-way variance (ANOVA) followed by a Bonferroni post-hoc test. Data were analyzed by SPSS (IBM SPSS Statistics v19.0), all statistical figures were created using GraphPad Prism 6.0.
